# First successful birth of twins in India following the transfer of vitrified oocytes

**DOI:** 10.4103/0974-1208.63124

**Published:** 2010

**Authors:** Priya Selvaraj, Kamala Selvaraj, Kalaichelvi Srinivasan

**Affiliations:** Department of Reproductive Medicine, Fertility Research Centre, GG Hospital, Chennai - 600 034, Tamilnadu, India

**Keywords:** Donor oocyte program, embryo transfer, ICSI, oocyte cryopreservation, vitrification

## Abstract

We report the first twin pregnancy and birth in India after the transfer of embryos generated from vitrified and thawed oocytes to a 41-year-old woman who was in a donor program. Embryos were generated from the microinjection of pre-prepared sperms into vitrified oocytes. Twin male babies weighing 750 and 860 g were born by emergency cesarean section. Vitrification is one of the most promising options in cryopreservation and preservation of embryos and oocytes.

## INTRODUCTION

Oocyte cryopreservation has recently gained more attention and became an integral part of assisted reproduction. An ultra-rapid freezing method called vitrification has been developed and it is a promising novel technique that requires no programmable freezing equipment. Vitrification is an efficient, fast, and economical method for oocyte cryopreservation providing a new alternative for the management of female infertility and fertility preserving options.

The technique involves placing the oocyte (preferably in its metaphase II stage) in a very small volume of vitrification medium and is then cooled at an extremely rapid rate. The fast freezing eliminates the formation of disruptive ice crystals within the oocyte. Recent advances in vitrification techniques have markedly improved the survival rate of oocyte after warming, and the pregnancy rates are being reported with greater frequency.[1–6] The most commonly accepted cryoprotectant for the vitrification procedure is ethylene glycol (EG) and sucrose both of which appear to have low toxic effects on embryos.[7,8] Pregnancies and normal live births achieved with vitrified embryos in humans suggest that EG is a good cryoprotectant for human vitrification.[9] The present study reports the first twin birth in India following vitrification with the transfer of viable embryos to a 41-year-old recipient in a donor program.

## CASE REPORT

Mrs. S, a known poor responder, aged 41 years with a history of previous failed attempts of IUI, enrolled with us for the donor program on 10^th^ November, 2008. The husband's semen parameters revealed oligoasthenozoospermia. Earlier, one treatment cycle had been cancelled owing to nonavailability of a suitable donor. She otherwise had no known medical or surgical illness. In her case, frozen-thawed oocytes were used because a suitable donor was unavailable for fresh donation and transfer. The couple was then counseled about the use of frozen-thawed oocytes and the expected low success rate for this cycle after obtaining informed consent.

**Figure 1:**
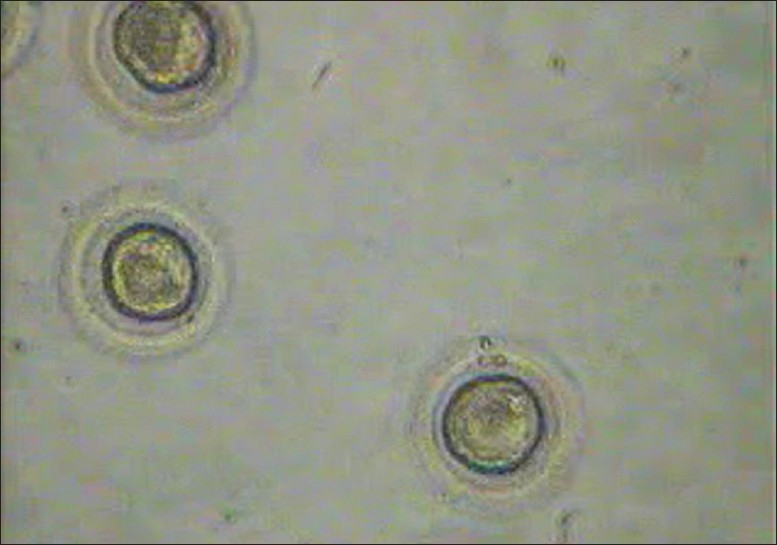
Mature oocytes

**Figure 2:**
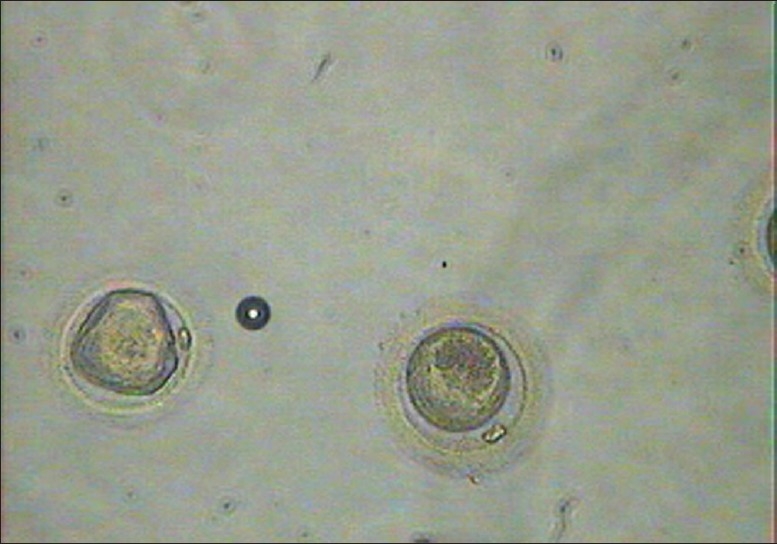
Mature oocytes suspended in Quinn's vitrification media

Vitrification was initiated 1-2 hr postretrieval after confirming that the oocytes are at metaphase II by denuding the cumulus with hyaluronidase (SAGE media, Trumbull CT, USA) and confirming metaphase II oocytes. Quinn's vitrification media (SAGE media, Trumbull CT, USA) was used to vitrify oocytes. Briefly, the oocytes were suspended in an equilibration solution containing 7.5% (v/v) ethylene glycol + 7.5% (v/v) dimethyl sulphoxide (DMSO) supplemented with human serum albumin (HSA) for 10 minutes at room temperature [Figures 1 and 2]. After equilibration, the oocytes were passed through four drops of vitrification solution (VS) containing 15% (v/v) ethylene glycol + 15% (v/v) DMSO + 0.6M sucrose supplemented with HSA. Suspension time in drops 1 and 2 was for 5 sec followed by 10 sec in drops 3 and 4. The loading from drop 4 was done in the allotted time of 90-110 sec. The oocytes were loaded into a specially designed vitrification device, the cryolock (Biodiseno Ltd., USA) in <1 μl of VS and directly plunged into liquid nitrogen (LN_2_) and transferred to the allotted Dewar of LN_2_.

**Figure 3:**
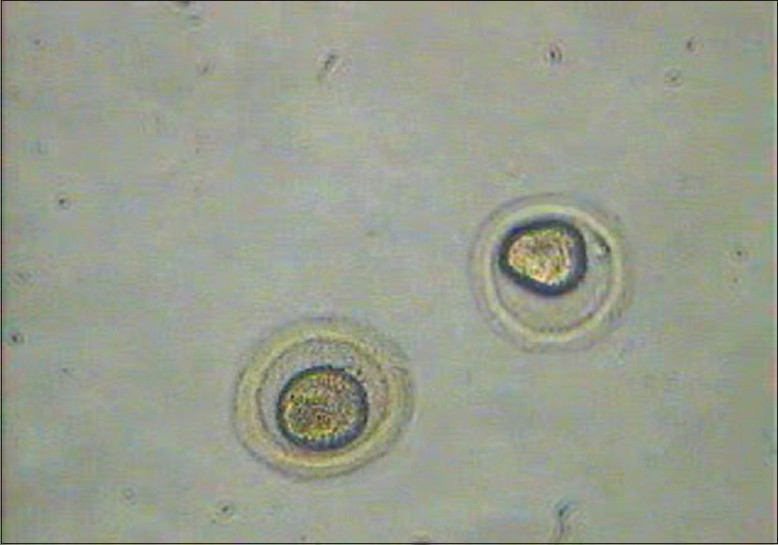
Thawed oocytes (first 3 minutes in MOPS)

**Figure 4:**
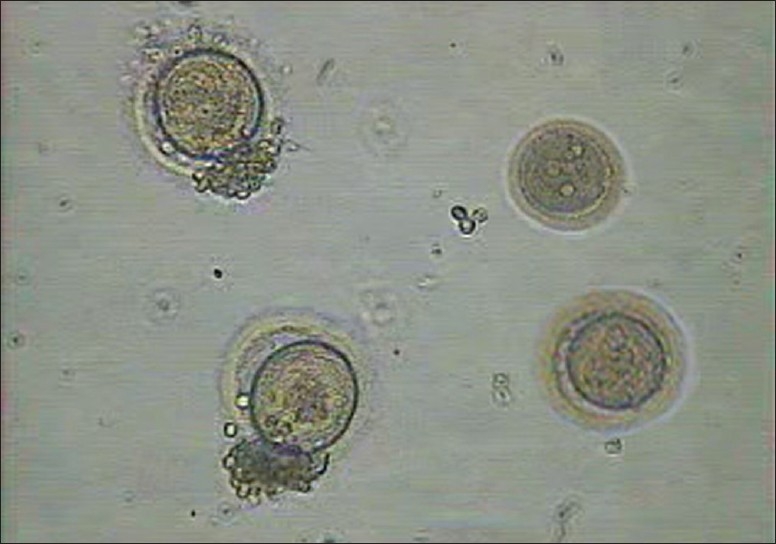
Thawed oocytes (last 3 minutes in MOPS)

**Figure 5:**
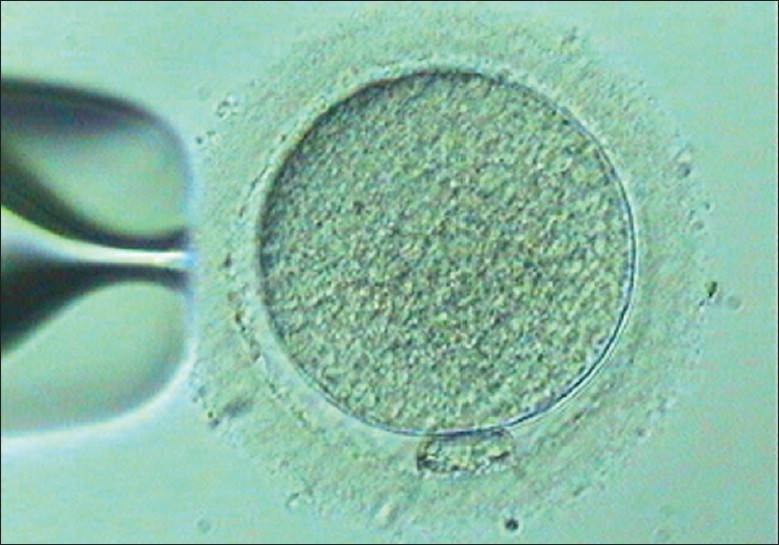
Holding egg

**Figure 6:**
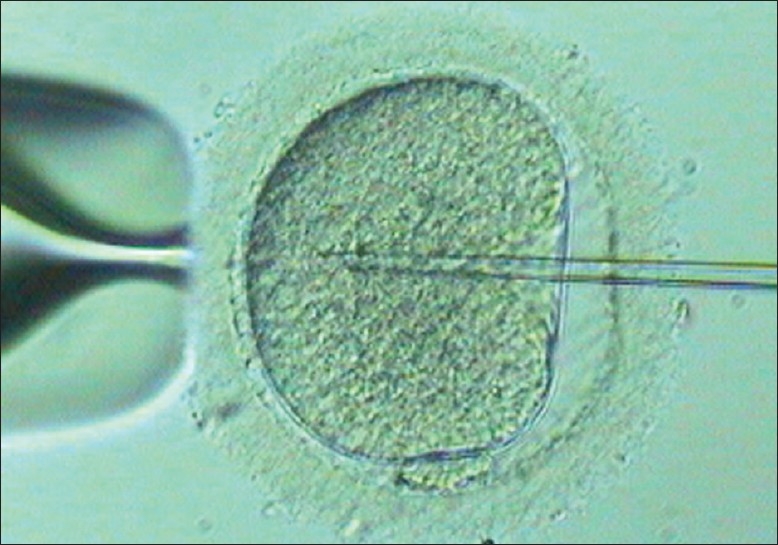
ICSI

Since our patient's parameters like blood group, eye color, hair color, physical characteristics including blood reports, personal habits, and genetic history were compatible with oocytes frozen by the above method from a potential donor 36 hr earlier, we thawed the same for her use. The endometrium was prepared with incremental doses of estrogen (estradiol valerate 2 mg, German Remedies, Mumbai, India) commencing from day 2 or day 3 of the cycle to a maximum of 8 mg/day with the initiation of micronized progesterone 400 mg thrice a day, from the day prior to warming. The optimum endometrium for transfer was considered to be 10 mm, which was observed in mid-cycle. Out of the 10 oocytes frozen by vitrification, we decided to thaw 6 oocytes. For the warming process, vitrification warming kit (SAGE media, Trumbull CT, USA) was used. The warming and dilution procedure were performed at 37°C. The cryolock devices were removed from LN_2_ Dewars and immediately immersed into the warming solution containing MOPS buffer supplemented with decreasing concentration of sucrose (1.0-0.5 mol/l) and HSA for 1 and 2 minutes, respectively. Then the oocytes were transferred to 3 drops of MOPS solution containing only buffer supplemented with HSA for 3 minutes each [Figures 3 and 4]. All the recovered oocytes were observed for parameters indicating good survival namely intact zona, clear perivitelline space, and visible polar body. A stippled appearance of ooplasm was deemed unfit. However, all six oocytes conformed to the above criteria, and they were further cultured in cleavage media (Quinn's SAGE *in vitro* fertilization, Trumbull CT, USA) at 37°C and 5% CO_2_ for 2 hr prior to ICSI. Viable oocytes were microinjected with the husband's sperms prepared by density gradient technique [Figures 5 and 6].

**Figure 7:**
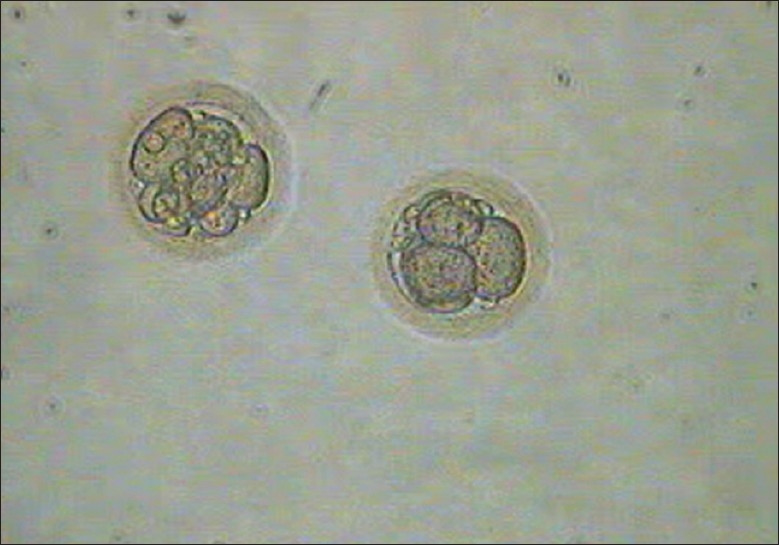
Embryos

**Figure 8:**
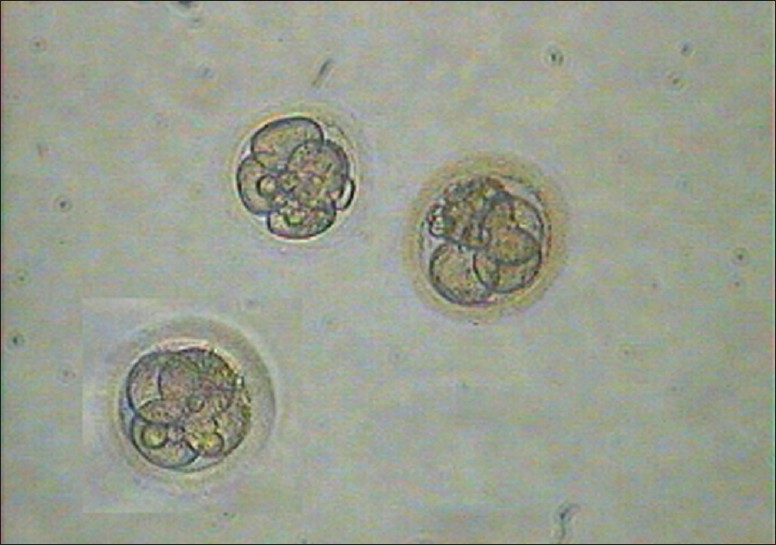
Embryos

The injected oocytes were further cultured in cleavage media in a trigas (5% CO_2_, 5% O_2_, and 90% N_2_) incubator until pronucleus check 16-18 hr later. Five embryos [1 (4 cells) grade I-II, 3 (4 cells) grade II, and 1 (5-6 cells) grade II][10] [Figures 7 and 8] were observed (day 2) and considering the expected low success rate with frozen thawed oocytes, all the five embryos were transferred on day 2 using a Labotect catheter (Labotect GmbH, Gottinggen, Germany). The serum β-hCG was done on 31^st^ March, 2009 and 2^nd^ April, 2009, which showed 159.4 and 588.5 mIU/ml, respectively. An ultrasound done on 38^th^ day revealed triplet gestational sacs, which eventually underwent spontaneous reduction to twins at 7 weeks. The first trimester progressed well with initial screening turning out to be normal. However, the patient became hypertensive at 22 weeks and was treated with conventional anti-hypertensives and stringent monitoring.

**Figure 9:**
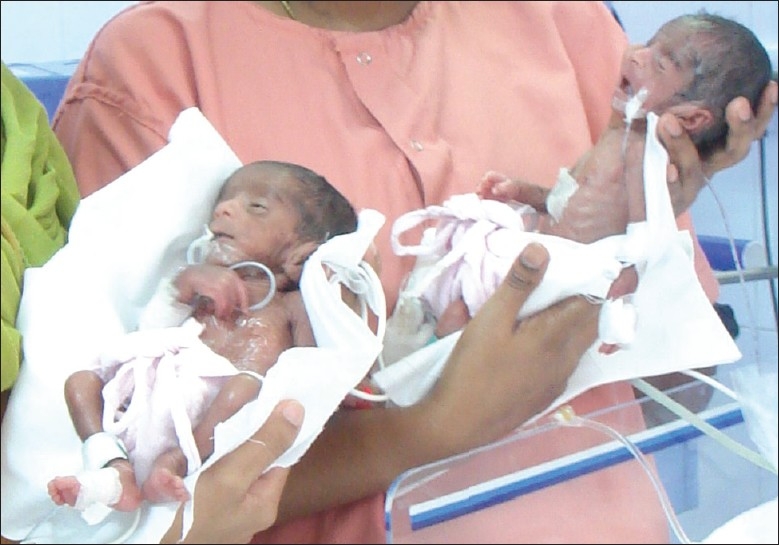
Twin I and II

Level II scan (systematic scan) was done on 6^th^ August, 2009, which was also normal, but signs of early growth restriction prompted us to admit her at 24 weeks for a closer follow-up. An emergency cesarean section was performed on 21^st^ September, 2009 at 29 weeks owing to a reversal of umbilical Doppler blood flow. Twin male babies weighing 750 and 860 g were delivered and initiated on ventilatory support [Figure 9]. One twin was extubated on 1^st^ October, 2009, while the other twin was extubated on 5^th^ October, 2009. Both the babies are doing well till date.

## DISCUSSION

Vitrification of gametes is a potentially less damaging procedure than the conventional slow freeze because it reduces intracellular ice formation and damaging osmotic effects that can occur during cooling and warming. This may be due to the rapid exposure to very high concentration of cryoprotectants in a minimal exposure time. In the initial phase of vitrification trials at our center with Irvine scientific media, oocytes were frozen at the germinal vesicle and metaphase I stages with no fertilization occurring after *in vitro* maturation and ICSI. Improving the survival rate and fertilization rate remains a major challenge in oocyte cryopreservation technology.[11] Currently we are using Quinn's vitrification media.

Vitrified oocytes have now been known to tolerate the osmotic stress in 0.5 M concentration of sucrose.[7] Oocyte freezing had met little success because of the low survival and fertilization rates.[12] Our data support this, but a long-term study is needed to improve learning curves and skills and also accurately determine what suits the laboratory and personnel to reproduce success. The survival rate of our vitrified oocytes was 63.9% and the fertilization rate with ICSI was 55.5% [Table 1].

**Table 1:** Statistics from November 2005 to October 2009

Total statistics	Total (%)	Slow freezing (%)	Rapid freezing (%)
No. of oocytes frozen	406	307	99
No. of oocytes thawed	144	96	48
No. of oocytes retrieved	132	96	36
No. of oocytes that survived the thawing process	85 (64.39)	62 (64.58)	23 (63.88)
No. of oocytes for which ICSI was performed	80	62	18
Total no. of oocytes fertilized	52 (65.00)	42 (67.74)	10 (55.55)
No. of cases for embryo transfer	13	10	3
No. of pregnancies	4 (30.76)	3 (30.00)	1 (33.33)
Preclinical	1	1	-
Delivered	2	1	1
Ongoing	1	1	0

## CONCLUSION

Vitrification seems to be a highly valuable tool in preserving human oocytes and it reduces the potential toxic effect. It also consumes less time, and it is cost effective with minimal use of equipments. Oocyte freezing is more feasible for the patients those with personal, moral, or religious objections to the cryopreservation of embryos which would eliminate the ethical and legal implications. It is much easier to discard frozen eggs instead of frozen embryos. Although applicable currently to certain European countries, it still holds good for universal application.

Optimization of the oocyte freezing system by the use of vitrification significantly contributes in improving the efficacy of various fertility preserving programs, such as oocyte banking and ovum donation.[13]

In the light of our success, following the announcement of first birth in India following oocyte cryopreservation (slow-freezing method), to our knowledge, this is the first twin birth in our country achieved after the transfer of vitrified oocytes. The opportunity to explore the response of human oocytes to vitrification in the present report opens a new frontier in assisted reproduction in our center and we believe it may be worth undertaking further research on this technique to determine the likely benefits for patients.
